# The effects of negative life events on depression in middle-aged women: the mediating role of rumination and coping styles

**DOI:** 10.3389/fpsyg.2026.1723958

**Published:** 2026-06-02

**Authors:** Yue Zhang, Xinyue Wang, Haonan Yu, Hongyan Jiang

**Affiliations:** Department of Psychiatry, First Affiliated Hospital of Kunming Medical University, Kunming, Yunnan, China

**Keywords:** chain mediation model, depression, middle-aged women, negative life events, rumination

## Abstract

**Background:**

The mental health of middle-aged women is a significant public health issue. Based on Response Styles Theory (RST), this study aims to investigate the associations between negative life events (NLEs), rumination, coping styles, and depression among middle-aged Chinese women, and to explore the chain mediation mechanism of rumination and positive coping styles.

**Methods:**

A cross-sectional survey was conducted among 327 middle-aged women aged 35–55 (case group: *n* = 157; control group: *n* = 170). Assessment tools included the Self-Rating Depression Scale (SDS), Life Events Scale (LES), Ruminative Response Scale (RRS), Simplified Coping Style Questionnaire (SCSQ), and Pittsburgh Sleep Quality Index (PSQI). Data were analyzed using SPSS 25.0, and the chain mediation effect was tested using the PROCESS macro.

**Results:**

(1) The case group scored significantly higher than the control group on depression and sleep disturbance. (2) Correlation analysis showed that NLEs, rumination, and positive coping styles (negatively) were all significantly associated with depression and sleep quality (*p* < 0.01). (3) The chain mediation model revealed that NLEs significantly predicted depression (*β* = 0.50, *p* < 0.001). Furthermore, NLEs were associated with depression through the chain mediation path of “Rumination → Positive Coping Style,” where increased rumination predicted decreased positive coping strategies.

**Conclusion:**

Negative life events are closely associated with decreased sleep quality and exacerbated depressive symptoms in middle-aged women. This relationship is mediated by the chain effect of “Rumination → Decreased Positive Coping Style,” suggesting that rumination may interfere with the adoption of positive coping mechanisms. Interventions should prioritize reducing rumination and enhancing positive coping strategies to alleviate depression and improve sleep quality.

## 1 Introduction

The mental health of middle-aged women (aged 35–55) has become a significant public health issue. Epidemiological data show that women have a significantly higher risk of developing depression than men, especially during this life stage (World Health Organization, 2017; Kuehner, 2017; GBD 2021 Mental Disorders Collaborators, 2024; Wu et al., 2023). Middle-aged women often find themselves under a “triple burden”: they not only need to balance the challenges of their professional careers but also bear the family responsibilities of raising children and caring for aging parents (Bower et al., 2020; Pope et al., 2012). These multiple and conflicting role demands can easily deplete their emotional resources and energy, posing a severe challenge to their mental well-being (Lorant et al., 2003; Chen and Zhou, 2022).

Negative Life Events (NLEs) are a core indicator for measuring an individual’s stress load (Greenhaus and Beutell, 1985; Williams, 2003; Choi and Marks, 2008; Tolin and Foa, 2006; Berent-Spillson et al., 2017; Cerrato and Cifre, 2018). They are defined as events that substantially restructure an individual’s life within a specific period, such as family conflicts, job changes, or the death of a loved one (Holmes and Rahe, 1967). Studies indicate that among the adverse experiences encountered by middle-aged women, familial events frequently exhibit the highest prevalence (Woods and Mitchell, 1997; Gullickson, 1993). Extensive evidence highlights NLEs as a significant risk factor for psychopathological symptoms, showing a close association with depression (Knight, 2017; Bağcaz and Kılıç, 2024), and anxiety (Giorgi et al., 2015), and serving as a strong predictor of sleep disturbances (Morin, 2022). Therefore, it is crucial to conduct an in-depth investigation into the associations between NLEs, depression, and sleep quality in this vulnerable demographic.

To understand the internal psychological mechanisms linking NLEs to depressive symptoms, the present study is anchored in the Response Styles Theory (RST) proposed by Nolen-Hoeksema (1991). RST suggests that how individuals respond to their negative emotions significantly predicts the severity and duration of depressive episodes. A core component of RST is rumination-a repetitive and persistent pattern of focusing on negative events or emotions rather than actively seeking solutions. According to RST, rumination is not only a common predictor of depression and anxiety (Nolen-Hoeksema, 1991), but also a key cognitive mechanism that amplifies negative emotional states. Crucially, RST posits that rumination consumes limited cognitive resources (Olatunji et al., 2013), thereby interfering with instrumental behavior and effective problem-solving abilities.

In the context of coping with stress, an individual’s coping styles play a vital regulatory role (Lazarus and Folkman, 1987; Carver et al., 1989). Positive coping styles, such as active problem-solving and seeking social support, are generally associated with lower levels of psychological distress. However, driven by the mechanisms outlined in RST, when individuals-particularly women-engage in severe rumination following NLEs, their capacity to initiate these protective, positive coping strategies is significantly suppressed. Instead of actively addressing the stressors, the cognitive depletion caused by rumination limits their behavioral repertoire, resulting in a marked decrease in positive coping (Tamres et al., 2002; Aldao et al., 2010). This lack of positive coping has been consistently correlated with exacerbated symptoms of depression and disrupted sleep rhythms (Harvey, 2002).

While current research has established the correlation between adverse life events and depression, the majority of studies exploring rumination and coping mechanisms have concentrated on adolescents (Robinson and Alloy, 2003). At present, there is an absence of systematic, theory-driven empirical research examining the internal mechanisms by which NLEs interact with the sequential pathway of rumination and positive coping styles among middle-aged Chinese women.

Based on Response Styles Theory and existing empirical evidence, this study aims to investigate the chain mediating role of rumination and positive coping styles in the association between NLEs and depression. We propose the following hypotheses:

*H1*: Middle-aged women in the case group will score significantly higher in depression, sleep disturbances, negative life events, and rumination, and score significantly lower in positive coping styles compared to the control group.

*H2*: Rumination mediates the relationship between negative life events and depression.

*H3*: Positive coping style mediates the relationship between negative life events and depression.

*H4*: Rumination and positive coping style sequentially mediate the relationship between negative life events and depression (i.e., NLEs are associated with increased rumination, which in turn predicts decreased positive coping styles, ultimately correlating with higher levels of depression).

## 2 Literature review

### 2.1 Negative life events

#### 2.1.1 Negative life events and middle-aged women

Negative life events (NLEs) refer to occurrences that substantially restructure an individual’s life pattern within a certain period, inducing significant changes in objective life structures such as social roles or environmental adaptation (Dohrenwend and Dohrenwend, 1974). For middle-aged women, recent literature highlights a unique cluster of chronic and acute NLEs associated with the “sandwich generation” phenomenon. These women frequently encounter multifaceted stressors, including caring for aging parents, managing adolescent children, and navigating career bottlenecks (Frone et al., 1992).

The association between NLEs and compromised mental health has been robustly documented. Extensive empirical evidence indicates that major stressful life events are significant risk factors for the onset of depressive episodes (Kendler et al., 1999). Moreover, recent studies conducted between 2020 and 2024 further emphasize that the cumulative stress from recurring familial and occupational NLEs is strongly correlated with elevated anxiety levels and significant sleep disturbances (Drentea, 2000; Li et al., 2019). However, while the direct correlation between NLEs and depression is well-established, the specific cognitive and behavioral mechanisms bridging this relationship require deeper theoretical exploration.

### 2.2 Rumination

#### 2.2.1 A cognitive vulnerability based on response styles theory

To understand how NLEs translate into depression, this study adopts the Response Styles Theory (RST) developed by Nolen-Hoeksema (1991). RST posits that an individual’s cognitive response to distress dictates the severity and chronicity of their depressive symptoms. A central construct within RST is rumination, defined as a repetitive and persistent pattern of self-focused thinking about negative events, emotions, and their potential implications, without translating these thoughts into active problem-solving (Nolen-Hoeksema and Morrow, 1991).

According to RST, rumination acts as a critical cognitive vulnerability. When middle-aged women experience NLEs, those who engage in rumination tend to amplify their negative emotional states. Recent meta-analyses confirm that rumination is a robust predictor of both depressive and anxiety disorders (Singh and Mishra, 2023). Furthermore, intrusive ruminative thoughts triggered by stressful events are strongly associated with prolonged sleep onset latency and impaired sleep quality, as the cognitive arousal prevents physiological relaxation (Carney et al., 2013).

### 2.3 Coping styles

#### 2.3.1 Positive coping styles and cognitive depletion

Coping styles reflect the dynamic regulatory processes through which individuals use cognitive and behavioral strategies to mitigate the impact of stress. Positive coping styles-such as active problem-solving, cognitive reappraisal, and seeking social support-are generally considered protective factors against psychopathology (Paul and Moser, 2009).

However, the effective deployment of positive coping strategies requires significant cognitive resources. Drawing upon RST and resource depletion models, persistent rumination consumes the limited executive functions and working memory required for constructive planning (Nolen-Hoeksema, 1991). When middle-aged women are overwhelmed by ruminative thoughts following NLEs, their cognitive bandwidth is occupied by the problem’s symptoms rather than potential solutions. Consequently, this cognitive depletion inhibits their ability to initiate positive coping behaviors. Research indicates that a reduced reliance on positive coping strategies correlates strongly with the accumulation of negative emotions, thereby exacerbating depressive symptoms and disrupting normal sleep patterns (Baglioni et al., 2011).

### 2.4 The relationship between negative life events, rumination, coping styles, and depression

#### 2.4.1 Theoretical justification for the chain mediation model

While previous research has tentatively explored the mediating roles of rumination or coping mechanisms individually, these studies have predominantly focused on adolescents or clinical populations, often treating these mediators in isolation (Park et al., 2022; McLaughlin et al., 2007). The current study addresses this gap by proposing an integrated chain mediation model specifically tailored to middle-aged women, theoretically grounded in RST.

We hypothesize a sequential pathway: NLEs do not merely exert a direct effect on depression; they also trigger a cascade of cognitive and behavioral dysregulations. First, NLEs activate rumination (the cognitive response). Subsequently, the excessive cognitive load imposed by rumination interferes with the individual’s capacity to engage in proactive, problem-solving behaviors, leading to a significant decrease in positive coping styles (the behavioral consequence). Finally, this lack of protective positive coping leaves the individual vulnerable to escalating depressive symptoms and diminished sleep quality.

This theoretical chain (NLEs → Increased Rumination → Decreased Positive Coping Style → Depression/Sleep Disturbance) provides a comprehensive framework to understand the psychological deterioration in middle-aged women, shifting the focus from simple variable associations to dynamic, theory-driven mechanisms.

## 3 Methods

### 3.1 Participants

This study was approved by the Ethics Committee of the First Affiliated Hospital of Kunming Medical University (Approval No. [2024] L-61), and all participants signed an informed consent form.

The study used a convenience sampling method, recruiting a total of 327 middle-aged women aged 35–55. Among them, 157 were in the case group, sourced from patients who visited the psychiatric outpatient and inpatient departments of the First Affiliated Hospital of Kunming Medical University between 2023 and 2024. The control group consisted of 170 healthy women from various communities and schools in Kunming during the same period. After data collection, a total of 4 invalid questionnaires were excluded due to missing or carelessly filled-in responses.

#### 3.1.1 Inclusion and exclusion criteria for the case group

Inclusion Criteria:

1. Women aged 35–55.
2. Education level of primary school or above, able to understand the test content and cooperate in its completion.
3. Voluntarily participated in the assessment and signed the informed consent form.
4. Participants met ICD-10 criteria for depressive disorder diagnosed by two senior psychiatrists.

Exclusion Criteria for the Case Group:

1. Physical health problems (such as severe neurological, endocrine, cardiovascular diseases, etc.).
2. Long-term alcoholism, drug addiction, alcohol abuse, etc.
3. Presence of self-harm or suicidal behavior.
4. History of schizophrenia, bipolar disorder, personality disorder, or being in an acute phase of the illness.

#### 3.1.2 Inclusion and exclusion criteria for the control group

Inclusion Criteria:

1. Women aged 35–55.
2. Education level of primary school or above, able to understand the test content and cooperate in its completion.
3. Voluntarily participated in the assessment and signed the informed consent form.

Exclusion Criteria:

1. Physical health problems (such as severe neurological, endocrine, cardiovascular diseases, etc.).
2. Long-term alcoholism, drug addiction, alcohol abuse, etc.
3. Presence of self-harm or suicidal behavior.
4. History of schizophrenia, bipolar disorder, personality disorder, or being in an acute phase of the illness.

### 3.2 Measurement tools

#### 3.2.1 Self-rating depression scale (SDS)

Depressive symptoms over the past week were assessed using the 20-item SDS (Zung, 1965). Responses are rated on a 4-point Likert scale, with higher standardized scores indicating more severe depressive tendencies (Wang and Wang, 1999). Cronbach’s *α* for the SDS in this study was 0.903.

#### 3.2.2 Simplified coping style questionnaire (SCSQ)

The revised SCSQ (Xie, 1998) was utilized to evaluate individuals’ coping strategies. While the scale measures both positive and negative coping, in alignment with our theoretical framework (Response Styles Theory) emphasizing the depletion of active problem-solving, only the positive coping dimension (e.g., problem-solving, seeking support) was utilized in the mediation model. Higher scores indicate a greater tendency to use positive coping strategies. Cronbach’s *α* for the positive coping subscale in this study was 0.875.

#### 3.2.3 Pittsburgh sleep quality index (PSQI)

Subjective sleep quality over the past month was measured using the Chinese version of the PSQI (Buysse et al., 1989; Liu et al., 1996). It yields a global score ranging from 0 to 21, with a total score of > = 7 indicating significantly impaired sleep quality (Zhang et al., 2019). Cronbach’s *α* in this study was 0.817.

#### 3.2.4 Life events scale (LES)

The Chinese version of the LES (Yang and Zhang, 1993) was used to quantify the cumulative psychological stress from 48 localized life events across family, work/study, and social domains. Higher weighted scores represent a more significant negative impact and potential harm to physical and mental health.

#### 3.2.5 Ruminative response scale (RRS)

Rumination was assessed using the 22-item Chinese version of the RRS (Nolen-Hoeksema, 1987; Han and Yang, 2009). It is rated on a 4-point Likert scale. Higher scores reflect more severe rumination. Cronbach’s *α* in this study was 0.968.

### 3.3 Data analysis

All collected data were analyzed using SPSS 25.0, with *p* < 0.05 set as the threshold for statistical significance (Faul et al., 2007; Preacher and Hayes, 2008). Descriptive statistics, independent samples t-tests, and chi-square tests were employed to compare demographic variables between the case and control groups.

Prior to mediation analysis, several critical statistical assumptions were evaluated. To address the reviewer’s concern regarding multicollinearity, the Variance Inflation Factor (VIF) and Tolerance values were calculated for all predictors. All VIF values were well below the threshold of 5 (ranging from 1.12 to 2.45), and Tolerance values were >0.2, indicating that multicollinearity was not a significant issue.

To address the potential socioeconomic confounding inherent in our sampling strategy (where clinical groups naturally exhibit lower employment and marital stability due to the functional impairment of depression), variables with significant group differences (education level, marital status, employment status, and having children) were strictly entered as covariates in all regression models. This allowed us to isolate the psychological mechanisms from the socio-economic “drift” often associated with clinical depression.

The mediation effect analysis was conducted using the PROCESS macro (Model 6) developed by Hayes. Model 6 was specifically selected because it is theoretically designed to test sequential chain mediation (i.e., X → M1 → M2 → Y), which perfectly aligns with our hypothesized pathway (NLEs → Rumination → Positive Coping → Depression). To ensure the robustness of the results (robustness checks), we employed the bias-corrected non-parametric percentile Bootstrap method with 5,000 resamples. An indirect effect was considered statistically significant if its 95% Confidence Interval (CI) did not include zero. Effect sizes for the mediation paths were also calculated to determine the practical significance of the model.

## 4 Results

### 4.1 Common method bias test

To address the potential common method bias (CMB) arising from self-report measures, procedural remedies such as reverse-scored items and anonymous administration were first implemented to minimize participant response sets. Subsequently, Harman’s single-factor test was conducted to statistically evaluate the data. The unrotated exploratory factor analysis revealed 8 factors with initial eigenvalues greater than 1. The first principal factor accounted for 34.50% of the total variance, which is well below the critical threshold of 40%. This indicates that there is no significant common method bias in the present study.

### 4.2 Comparison of demographic characteristics and study variables

Independent samples t-tests and chi-square tests revealed no statistically significant differences between the case and control groups regarding age, ethnicity, and place of residence (*p* > 0.05). However, significant group differences were observed in educational level (*p* = 0.02), marital status (*p* < 0.01), employment status (*p* < 0.01), and having children (*p* = 0.02). Specifically, the case group had higher proportions of participants with a primary school education, who were unmarried/divorced, unemployed, and childless. These variables were included as covariates in the subsequent regression and mediation analyses.

Regarding the primary study variables, the case group scored significantly higher on depression, sleep disturbance, negative life events (NLEs), and rumination compared to the control group (all *p* < 0.01). Conversely, the case group scored significantly lower on positive coping style than the control group (*p* < 0.01). Detailed descriptive statistics and group comparisons are presented in Table 1.

**Table 1:** Comparison of demographic characteristics and study variables between case and control groups.

Variable	Group	*n*	Mean (SD)/*n* (%)	*χ*^2^/*t*	*p*
Demographics					
Age (35–41 years)	Case	56	35.67%	4.07	0.13
	Control	45	26.47%		
Age (42–48 years)	Case	49	31.21%		
	Control	53	31.18%		
Age (49–55 years)	Case	52	33.12%		
	Control	72	42.35%		
Education level (Primary school)	Case	23	14.65%	11.68	0.02*
	Control	11	6.47%		
Education level (Junior high school)	Case	39	24.84%		
	Control	41	24.12%		
Education level (Senior high school)	Case	18	11.46%		
	Control	38	22.35%		
Education level (Junior college)	Case	24	15.29%		
	Control	30	17.65%		
Education level (University and above)	Case	53	33.76%		
	Control	50	29.41%		
Marital status (Unmarried)	Case	11	7.01%	13.26	<0.01
	Control	3	1.76%		
Marital status (Married)	Case	121	77.07%		
	Control	155	91.18%		
Marital status (Divorced)	Case	24	15.29%		
	Control	12	7.06%		
Marital status (Widowed)	Case	1	0.63%		
	Control	0	0.00%		
Employment status (Employed)	Case	113	71.97%	11.53	<0.01
	Control	148	87.06%		
Employment status (Unemployed)	Case	44	28.03%		
	Control	22	12.94%		
Study variables					
Depression	Case	157	63.31 ± 8.23	22.86	<0.01
	Control	170	43.34 ± 7.56		
Sleep quality	Case	157	11.89 ± 2.11	16.06	<0.01
	Control	170	5.32 ± 1.87		
Negative life events	Case	157	18.41 ± 7.62	6.80	<0.01
	Control	170	11.23 ± 5.15		
Rumination	Case	157	52.24 ± 10.33	11.76	<0.01
	Control	170	40.45 ± 9.56		
Positive coping style	Case	157	13.52 ± 4.15	−17.76	<0.01
	Control	170	24.38 ± 5.12		

### 4.3 Correlation analysis of major study variables

Pearson correlation analysis was conducted to examine the linear associations among the core variables. As shown in Table 2, significant correlations were found among all study variables. Specifically, NLEs were positively correlated with rumination (*r* = 0.60, *p* < 0.01), depression (*r* = 0.54, *p* < 0.01), and sleep disturbance (*r* = 0.48, *p* < 0.01), and negatively correlated with positive coping style (*r* = −0.30, *p* < 0.01). Furthermore, positive coping style was negatively correlated with both rumination (*r* = −0.44, *p* < 0.01) and depression (*r* = −0.44, *p* < 0.01).

**Table 2:** Correlation coefficients between variables.

Variable	1	2	3	4	5
Negative life event	–				
Rumination	0.6^**^	–			
Coping style	−0.30^**^	−0.44^**^	–		
Depression	0.54^**^	0.71^**^	−0.44^**^	–	
Sleep quality	0.48**	0.59**	0.58**	0.58**	–

**p < 0.01, *p < 0.05.

### 4.4 Mediation effect analysis

To test the hypothesized chain mediation model (NLEs → Rumination → Positive Coping Style → Depression), Hayes’ PROCESS macro (Model 6) was utilized. The analysis controlled for demographic covariates (educational level, marital status, employment status, and having children).

The regression results (Table 3) showed that NLEs positively predicted rumination (*β* = 0.57, *p* < 0.01), but did not significantly predict positive coping style (*β* = −0.05, *p* > 0.05). Rumination negatively predicted positive coping style (*β* = −0.43, *p* < 0.01) and positively predicted depression (*β* = 0.53, *p* < 0.01). Positive coping style negatively predicted depression (*β* = −0.15, *p* < 0.01). NLEs remained a significant positive predictor of depression (*β* = 0.50, *p* < 0.01) even when the mediators were included in the model.

**Table 3:** Chain mediation model.

Predictor variable	Equation 1: rumination		Equation 2: positive coping style		Equation 3: depression		Equation 4: depression	
	*β*	*t*	*β*	*t*	*β*	*t*	*β*	*t*
Educational attainment	0.05^**^	3.58	0.24^**^	4.78	−0.02	−0.43	−0.01	−0.28
Marital status	0.07	1.00	−0.09	−1.80	0.11	2.14	0.05	1.27
Work or not	0.01	1.45	−0.08	−1.61	0.00	0.09	−0.01	−0.28
With children or not	0.11	2.28	−0.00	−0.01	0.19^**^	3.62	0.12^**^	2.85
Negative life event	0.57^**^	12.69	−0.05	−0.77	0.50^**^	10.61	0.15^**^	3.28
Rumination			−0.43^**^	−7.11			0.53^**^	10.36
Positive coping style							−0.15^**^	−3.49
*R^2^*	0.37		0.28		0.32		0.55	
*F*	*F*(5, 321) = 38.06 *p* = 0.00		*F*(6, 320) = 20.26 *p* = 0.00		*F*(5, 321) = 29.66 *p* = 0.00		*F*(7, 319) = 56.44 *p* = 0.00	

**p < 0.01, *p < 0.05.

**Figure 1:**
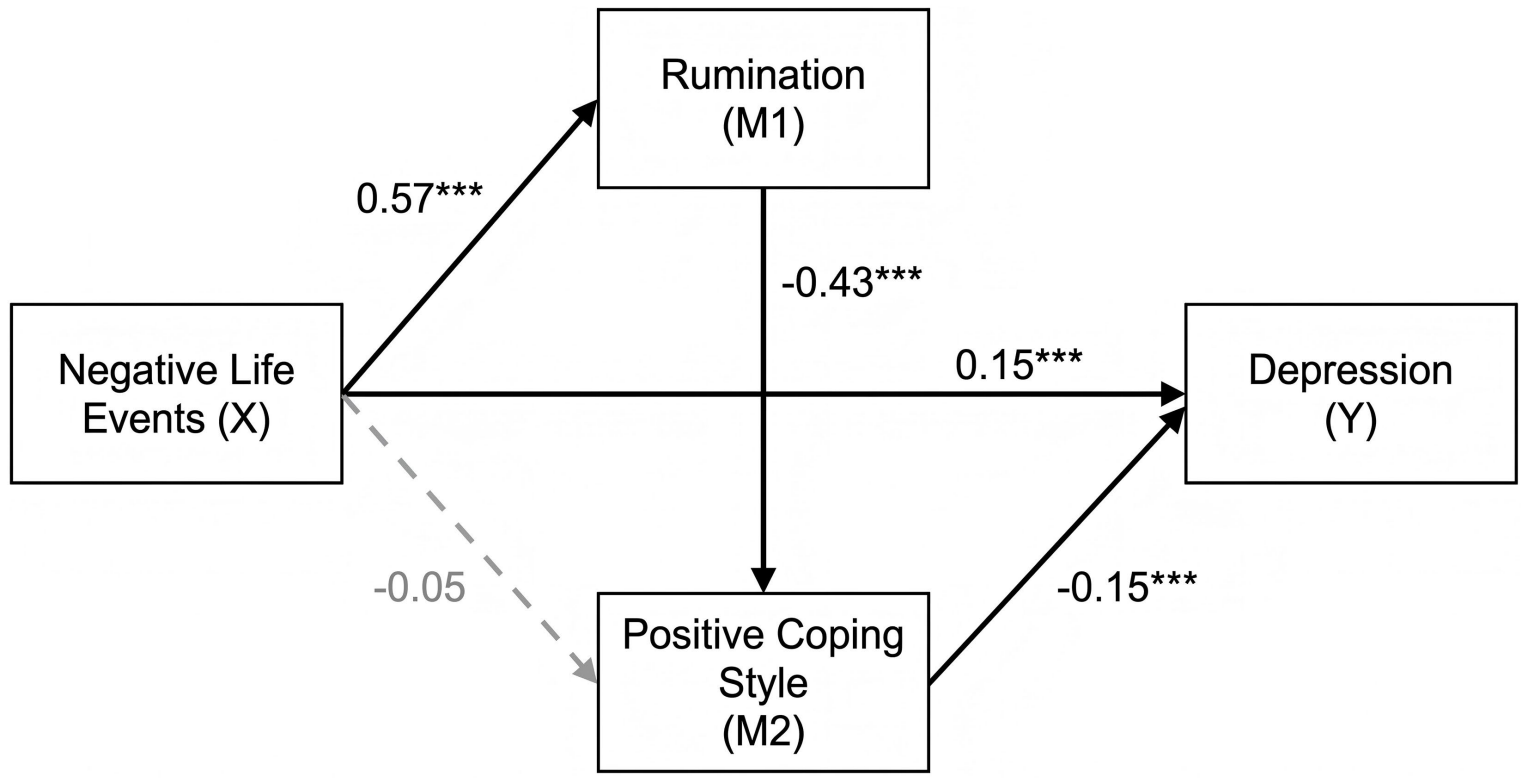
Chain mediation model. Solid lines indicate significant pathways (**p < 0.01, ***p < 0.001). Dashed lines indicate non-significant pathways. Values on the paths represent standardized coefficients (β).

The mediation effects were tested using 5,000 bootstrap samples (Table 4). The analysis identified three indirect pathways. The indirect effect of Pathway 1 (NLEs → Rumination → Depression) was significant (Effect = 0.101, 95% CI [0.079, 0.130]). The indirect effect of Pathway 2 (NLEs → Positive Coping Style → Depression) was not significant (Effect = 0.003, 95% CI [−0.002, 0.009]), as the confidence interval included zero. The indirect effect of the sequential Pathway 3 (NLEs → Rumination → Positive Coping Style → Depression) was significant (Effect = 0.011, 95% CI [0.005, 0.019]). The direct effect of NLEs on depression was 0.050 (95% CI [0.022, 0.079]) (Figure 1).

**Table 4:** Direct and indirect effects of the chain mediation model.

Model path	Effect value	S. E.	95%CI (Bootstrap)	
			Lower	Upper
Ind1	0.101	0.013	0.079	0.130
Ind2	0.003	0.003	−0.002	0.009
Ind3	0.011	0.004	0.005	0.019
Direct effect	0.050	0.015	0.022	0.079
Total	0.166	0.015	0.137	0.195

Ind1, Negative life events → Rumination → Depression.

Ind2, Negative life events →Positive Coping Style → Depression.

Ind3, Negative life events → Rumination → Positive Coping Style → Depression.

## 5 Discussion

### 5.1 General findings and implications

The present study investigated the complex mechanisms bridging negative life events (NLEs), cognitive-behavioral processes, and mental health outcomes (depression and sleep quality) in Chinese middle-aged women. Our findings reveal that the case group experienced significantly higher levels of NLEs, rumination, depression, and sleep disturbances, while reporting fewer positive coping strategies than the control group. Importantly, the structural equation model confirmed the hypothesized chain mediation effect (H4): NLEs predict increased rumination, which sequentially correlates with decreased positive coping styles, ultimately associated with exacerbated symptoms of depression (Greenhaus and Beutell, 1985; Williams, 2003; Choi and Marks, 2008; Tolin and Foa, 2006; Berent-Spillson et al., 2017; Cerrato and Cifre, 2018). This provides empirical evidence for applying Response Styles Theory (RST) to understand the psychological mechanisms specific to this high-risk demographic.

### 5.2 Independent mediation of rumination

Our results supported H2, indicating that rumination independently mediates the relationship between NLEs and depression. Grounded in RST, this finding highlights rumination as a critical cognitive vulnerability (Nolen-Hoeksema, 1991). Middle-aged women, often trapped in the “sandwich generation” dynamic, face chronic stressors from professional demands, caregiving for aging parents, and raising children (Bower et al., 2020). When faced with these NLEs, individuals prone to rumination enter a cycle of repetitive thinking about their distress, its causes, and consequences (Nolen-Hoeksema and Morrow, 1991).

This repetitive cognitive focus amplifies negative mood states rather than resolving the problems (Singh and Mishra, 2023). It also leads to cognitive arousal, interfering with physical and mental relaxation required for sleep onset (Carney et al., 2013). The high correlation found between rumination and sleep disturbance (*r* = 0.59) underscores this mechanism. Thus, our findings suggest that rumination is a key pathway linking external life stressors to the onset and maintenance of depressive symptoms.

### 5.3 The crucial link: depletion of positive coping via rumination

The most noteworthy contribution of this study lies in interpreting the chain mediation pathway (X → M1 → M2 → Y).

A critical detail in our regression analysis is that the direct effect of NLEs on positive coping style was not significant (*β* = −0.05, *p* > 0.05). This indicates that external stressors themselves do not directly reduce an individual’s adoption of active coping strategies (e.g., problem-solving, seeking support). Instead, the deployment of positive coping is contingent upon cognitive processing.

We interpret this finding through the lens of cognitive resource depletion models within the framework of RST (Koster et al., 2011). Effective problem-solving (positive coping) requires executive functions and working memory capacity (Davis and Nolen-Hoeksema, 2000; Muris et al., 2005; Nolen-Hoeksema et al., 2008; Koutsimani et al., 2019). Rumination, as an automatic, repetitive cognitive style, consumes significant amounts of these limited cognitive resources (Cohen, 1988). Therefore, when NLEs trigger severe rumination, middle-aged women become cognitively overloaded. This “cognitive bandwidth” depletion interferes with their ability to formulate proactive plans or seek support, leading to the observed decrease in positive coping (Baglioni et al., 2011). Consequently, this leaves them vulnerable to worsening depressive symptoms and further compromised sleep quality.

### 5.4 Cultural and developmental context

While maintaining caution regarding specific demographic structures not directly assessed in this study, our findings reflect the unique challenges faced by Chinese middle-aged women within modern society. The “sandwich burden” is intensified by the high cultural value placed on filial piety, demanding extensive commitment to elder care, simultaneously with intense parental commitment often required by competitive educational environments for their children. This combination of roles can easily trigger negative life events and increase vulnerability to psychological distress, particularly when rumination dominates as a response style.

### 5.5 Clinical implications

The findings of this study offer several critical clinical implications for addressing depression in middle-aged Chinese women. First, given the central role of rumination in the chain mediation model, clinical interventions should prioritize cognitive restructuring. Specifically, Mindfulness-Based Cognitive Therapy (MBCT) or Acceptance and Commitment Therapy (ACT) could be implemented. Empirical studies have demonstrated that mindfulness serves as a core protective factor that effectively mitigates negative emotions by significantly enhancing emotional resilience in Chinese populations (Wang et al., 2016). By fostering non-judgmental present-moment awareness, mindfulness practices can help these women recognize and disengage from the vicious cycle of rumination, thereby preventing their cognitive resources from being depleted by NLEs.

Second, considering that the case group exhibited significantly poorer sleep quality and higher anxiety levels, clinicians should adopt a multidimensional perspective when assessing depressive symptoms. Targeted interventions such as Cognitive Behavioral Therapy for Insomnia (CBT-I) or relaxation training may not only improve physiological sleep metrics but also indirectly weaken the influence of negative emotional states by enhancing overall psychological resilience. Finally, social support systems, including community-based counseling and family interventions, should identify high-risk women within the “sandwich generation” to facilitate a transition from emotional suppression toward more proactive, problem-oriented coping strategies.

### 5.6 Limitations and future directions

Several limitations must be acknowledged cautiously. First, as a cross-sectional study, it cannot infer definitive causal relationships between variables. Although grounded in theoretical models, future longitudinal or experimental designs are essential to explore the dynamic relationships between variables more deeply. Second, all measures were based on self-report instruments. While Harman’s single-factor test indicated no severe common method bias (CMB) in our data, the reliance on self-report questionnaires still poses risks of social desirability and recall bias. Future studies should consider incorporating multi-source data (e.g., informant reports or clinical objective evaluations) to improve data reliability.

Third, our sample was drawn primarily from middle-aged women seeking outpatient treatment in the Kunming area, and there were notable demographic differences between the case and control groups. Although these socio-economic variables were controlled for statistically, this convenience sampling limits the broader interpretability and generalizability of the findings across different regions and cultural backgrounds. Finally, our case group included patients at various clinical stages of depression (e.g., acute episodes vs. maintenance phases). This clinical heterogeneity may have introduced variance, particularly in rumination and sleep quality scores. Future research should stratify patients by illness stage to better control for these confounding effects.

## 6 Conclusion

This study reveals that among Chinese middle-aged women aged 35–55, negative life events affect depressive mood through the chain mediation pathway of “rumination → decreased positive coping style.” This not only validates the stress-cognition theory but also highlights the amplifying effect of rumination and the secondary depleting effect of reduced positive coping. The study localizes the cognitive-behavioral model to the highly vulnerable group of middle-aged women, providing a new theoretical perspective for understanding mental health issues at the intersection of gender and age-related stress.

## Data Availability

The raw data supporting the conclusions of this article will be made available by the authors, without undue reservation.
